# Impacts of the EU GMO regulatory framework for plant genome editing

**DOI:** 10.1002/fes3.161

**Published:** 2018-12-10

**Authors:** Penny A. C. Hundleby, Wendy A. Harwood

**Affiliations:** ^1^ John Innes Centre Norwich UK

**Keywords:** biotech crops, CRISPR, ECJ ruling, genetic modification, genome editing, GM trade, GMO regulation, NBTs

## Abstract

New plant breeding technologies, such as genome editing, are enabling new crop varieties to be developed far quicker and with greater precision and scope than achievable using conventional methods. These advances could help farmers address the challenges of climate change, sustainability, and global food security. However, despite their potential, the uptake of these new technologies has been slowed down due to the uncertainty associated with the regulation of genome edited crops. For many European consumers, their view of new breeding technologies is influenced by many factors. Those who have never faced a major food crisis may not sufficiently appreciate the challenges posed by a projected rise of 2 billion in the human population by 2050. In addition, consumers with a regular and plentiful supply of food may not have to consider how their food is produced, or appreciate the challenges EU farmers are already facing to meet future demand. Misleading online articles, questioning the safety and ethics of these “new” biotech foods, can also lead consumers to be reluctant to accept them. Consequently, Europe's mixed view on biotech crops may also be hindering their adoption in countries who have even more to gain from the technology. In this review, we discuss the current data on global and EU GM crop adoption and the potential impact a new wave of crop development may have for agriculture. We reflect on how the EU has viewed GM crops, and we consider the future of both genetic modification (GM) and genome editing (GE) in the EU. We explore lessons learnt from the adoption of GM crops and examine the potential impact the recent decision not to exempt genome edited crops from the EU GMO Directive, will have on EU farmers, scientists, consumers, trading countries, and the rest of the world.

## 1 GENETIC MODIFICATION—A TECHNOLOGY WITH MISSED OPPORTUNITIES IN EUROPE. AS EU MEMBER STATES CONTINUALLY FAILED TO REACH AGREEMENT

Genetic modification is defined here as the use of recombinant DNA technology to introduce genes directly into a plant's genome. These genes can be from within the same species or across species boundaries to produce novel crops. In the 22 years since GM, or biotech, crops have been commercially planted across the globe, and only two events have made it through the approval system for commercial cultivation in Europe. One event, an insect‐resistant maize, developed by Monsanto (called MON810) was approved in 1998. The other, a potato with altered starch qualities for industrial use, was developed by BASF (Amflora) and approved by the European Commission (EC) in 2010. BASF later withdrew Amflora from the EU market, citing a lack of market acceptance. The EC's decision to approve the event was also later questioned and taken to the European Court (case T‐240/10) who in 2013 annulled the approval, after it ruled the European Commission had failed to follow correct procedures. Adoption of MON810 by European farmers was also limited as many European Member States (Austria, Bulgaria, Germany, Greece, France, Hungary, Italy, Luxembourg, and Poland) effectively banned the cultivation of this approved crop on their territory, evoking “safeguarding clauses,” citing socio‐economic reasons, or applying emergency measures to prohibit cultivation of this crop. These restrictions were only intended to be temporary, while evidence was gathered in support of the claims. However, the European Food Safety Authority (EFSA) judged all applications of the safeguarding clauses to be scientifically unfounded (European Parliament, 2015; Qaim, 2016). As such, the continued restrictions were considered by some to be “illegal bans” jeopardizing internal markets and were later challenged in the European Court (Davison & Ammann, 2017; Kershen, 2014; Smart, Blum, & Wessler, 2015). While the EU has approved imports of maize containing the MON810 event, for food and feed use, support for its cultivation fell. Of the six EU countries who had previously planted MON810 (Germany, Poland, Slovakia, Romania, Czech Republic, Portugal, and Spain), only Spain and Portugal continued to grow it in 2017 (ISAAA, 2017).

The MON810 event, approved in 1998, would be unlikely to gain regulatory approval today, but not because the product is unsafe. EFSA's role is to evaluate all crops, putting forward *only* those that meet the strict biosafety criteria to the European Commission (EC). The EC's role is then to “draft a decision” to put to the Member States Expert Committee. To obtain regulatory approval for cultivation, an event requires a qualified majority vote in support of its cultivation by Member States (MS). The number of member states within the EU in 1998 was 14, whereas in 2018 there were 28, thus making it harder to reach a consensus. In cases where a qualified majority vote is not reached (be that in favor of approval, or a ban on cultivation), the European Commission convenes, requests further information from the applicant via EFSA, and then returns a revised opinion to the Appeal Committee (it was the ECs failure to submit a revised version to MS on the BASF Amflora case that resulted in the court annulling the ECs approval of this product). The Appeal committee are again required to reach a qualified majority vote in favor of adoption for authorization to be approved. When no opinion or qualified majority is reached by Member States, the decision passes back to the European Commission.

The renewal of MON810 (which is required every 10 years following approval) has remained “pending” since 2007 as MS continually fail to reach a majority vote, even though the product has been used safely for more than 20 years. While a renewal application is pending, authorization remains in place for its cultivation. In 2015, the “opt‐out” or Cultivation Directive was introduced. Its intention was to give MS opposed to GM cultivation the freedom to “opt‐out” of a developer's application for approval, by restricting or prohibiting the cultivation of the GM crop on their own territory, without the need for scientific justification, allowing individual MS values to be respected (DIRECTIVE (EU) 2015/412). However, in 2017 in the vote on the renewal of MON810 to the Appeal Committee, 14 MS still voted against the proposal, eight were in favor and six abstained (with a qualified majority not obtained) (EC, Comitology Register, 2017). The decision was again passed back to the European Commission, who granted the authorization for MON810 for feed and food use on 4 July 2017 (EC, 2017) but where it remains pending concerning the use of seed for cultivation.

## 2 WHILE EUROPE CONTINUES TO HOLD BACK—THE VALUE OF BIOTECH CROPS HAS BEEN REALIZED ELSEWHERE…

Meanwhile, other parts of the world have embraced and taken advantage of the technology. In 2017, commercial farmers in 24 (19 developing and five industrial) countries grew 189.8 million hectares of biotech crops with economically important traits such as insect resistance, herbicide tolerance, and stacks thereof with—disease resistance, drought tolerance, product quality traits such as anti‐allergy, delayed fruit softening, modified oil/fatty acid content etc., as well as pollination control traits (ISAAA, 2017). The average biotech crop adoption rate in the top five biotech crop‐growing countries also increased in 2017 to reach close to saturation, with the USA at 94.5% (average for soybeans, maize, and canola adoption), Brazil (94%), Argentina (~100%), Canada (95%), and India (93%) (ISAAA, 2017). The total number of countries consuming biotech crops reached 67 including 43 countries that do not cultivate GM crops themselves (17 + 26 EU countries). This means that in some countries, farmers are not permitted to cultivate these crops, but the same crops can be imported for food and feed markets!

A further consequence of the EU's attitude toward GM crop cultivation can be seen in the region's research. Historically at the forefront of GM crop research, the number of field trials in Europe has also been declining and decreased by 90% between 2010 and 2016, illustrating the negative trend for GM developments in Europe. Of the 28 MS, only 11 MS currently permit GM field trials, and only six MS conducted open‐field testing in 2017: Belgium, the Czech Republic, Romania, Spain, Sweden, and the United Kingdom (USDA Foreign Agricultural Service, Gain Report. EU‐28 AgBiotech [Link] report).

## 3 THE GM DEBATE—WHAT IMPACT DOES THE EU GM APPROVALS PROCESS HAVE ON EU FARMERS?

Compared to other major agricultural producers, such as China, the USA, and Brazil, overall agricultural growth in the EU has been stagnating over the last decade. This in part has been a result of farmers not having access to the same technologies and crop protection products as the rest of the world (AgbioInvestor report: Agricultural Market Intelligence). So while those opposed to biotechnology may regard the absence of GM in EU fields as a success, there has been frustration among scientists, plant breeders, and farmers who seek the opportunity to develop improved crops and the freedom to choose the best crops, be they GM or non‐GM, to meet their needs. For a GM crop to be cultivated (or imported) into the EU, developers are required to provide information and data to enable a full scientific safety assessment of the new crop by EFSA to show that such a crop poses no danger to human and environmental health. Only products that comply with the scientific safety criteria can then enter the voting system. Despite the favorable opinion of EFSA, a qualified majority MS vote in favor is still needed to proceed with the cultivation, or import, of that crop. This then turns the process from one of scientific assessment to a political or socio‐economic decision rather than one concerning safety, as the introduction of a biotech crop might lead to both socio‐economical changes and changes to agriculture and industry. Such changes do need to be considered alongside the scientific assessment. However, addressing the resulting ethical questions is fraught with difficulty and conflict. Various approaches for dealing with ethical questions surrounding the use of GM crops have been recently reviewed (Ricroch, Guillaume‐Hofnung, & Kuntz, 2018).

Protection of organic agriculture and problems of co‐existence with biotech crops is often cited as one of the key socio‐economic issues. European Commission statistics show that the organic sector now represents 6.2% of the agricultural area in Europe. However, the question of whether GM crops could also be organic is receiving renewed attention (Husaini & Sohail, 2018). Crops, which do not require chemical inputs, that have traits to mitigate some adverse effects of climate change and have beneficial nutritional qualities would certainly be welcomed in organic agriculture. Maybe it is time to look again at the middle ground between organic and conventional technologies, and how conservation farming views GM. Here, the imperative is to focus on protecting the environment, managing soils and farmland as an ecosystem. These benefits could be available to the organic sector should such crops be approved for cultivation in the EU in the future. However, many organic trade associations remain opposed to all GMOs (not just herbicide tolerant crops) and consider new plant breeding technologies as genetic modification techniques not compatible with organic farming (IFOAM Organics International, 2017).

There is evidence, however, that EU farmers are missing out on the economic benefits enjoyed by farmers in countries that have embraced GM crops (Brookes & Barfoot, 2018). Between 1999 and 2006, Romanian farmers had access to GM herbicide tolerant (HT) soybean, during which time they reported net gains of 18% due to better weed management and reduced inputs and Romania became a net exporter of soybean for the first time. However, in 2007 Romania joined the EU and was no longer permitted to grow GM HT soybean, as it was not approved for cultivation in the EU. As a result, within 2 years, the area planted to soybeans dropped by 70%, and Romania became a net importer of vegetable protein, just like the European Union itself. Romanian farmers were deprived of a unique opportunity to produce an export crop, lower the cost of their animal feed, and thus increase their competitiveness in the global marketplace (Otiman, Badea, & Buzdugan, 2008). To date, EU farmers are still not permitted to grow GM HT soybean, a crop which now accounts for more than 50% of all GM crops grown worldwide (ISAAA, 2017). Yet the EU imports over 19 million tonnes of soybean typically a year (EU benefits from GM trade, EuropaBio infographic), the majority of which is GM.

## 4 THE IMPACT OF ASYNCHRONIZED APPROVALS—AT WHAT POINT DOES IT BECOME UNSUSTAINABLE FOR CONVENTIONAL EU FARMERS TO COMPETE?

In the EU, 22 million farmers and agricultural workers are employed in the agri‐food sector, making it one of the most significant economic sectors. These farmers provide a stable food supply, for more than 500 million Europeans. The European Union's farm policy ensures a decent standard of living for farmers, at the same time as setting requirements for animal health and welfare, environmental protection, and food safety. However, based on the EU's own data, long‐term farming profitability is only currently attainable by financial support in the form of subsidy from the EU (AgbioInvestor report: Agricultural Market Intelligence).

As well as the frustration of farmers wishing to have access to GM technology, Europe also has one of the slowest approval times for GM imports entering the EU, with some products taking more than 10 years for approval behind their US counterparts (source (ISAAA GM approval database)). These delays result in a disruption of trade and limit the range of commodities European traders can select from. In cases like soybean, for which the global market share in 2017 was 77% GM (ISAAA, 2017), the main exporters to the EU (USA, Brazil, and Argentina) are no longer able to meet the EU's non‐GM demand, with more than 94% of the importer's soybeans being GM. With the EU meeting just 5% of its own soybean needs, this leaves the EU vulnerable from a food security point of view. These soybean imports are used mainly to feed livestock which is one of Europe's biggest exports. Lack of access to all GM events in a trader's catalogue will mean farmers potentially not getting the best financial deals. With farmers elsewhere having access to this technology, at what point does it become unsustainable for conventional EU farmers to compete? Yield losses and reduced quality due to poor weed control can have a significant economic impact. A study published in 2017, covering a 6‐year period in North America, showed weed interference in soybean fields caused a 52.1% yield loss (Soltani et al., 2017). Therefore, a conventional farmer may potentially need twice the land area and inputs to match the yields of farmers elsewhere, but is this competitive or even sustainable?

Such asynchronized regulatory systems also affect other countries too—a classic example of disrupted trade being that of the Hawaiian GM Papaya industry and Japan. The industry suffered lost trade of 15 million pounds of fruit, due to a 10‐year wait for Japan's approval to import their GM product (Gonsalves, 2014). For countries to have the confidence to embrace a new technology they need to be confident they can sell their crops both internally and across an external market. With the EU having such reluctance to embrace GM technology, and a slow approval process for imports, this must be a consideration for countries choosing to trade with the EU. There is also the economic burden for an exporting country to maintain strict segregation of commodity crops to ensure only EU approved GM events are imported, in the absence of other non‐approved GM events (safely traded elsewhere) but awaiting approval within the EU. This puts a financial burden on traders—a cost ultimately passed on to the consumer. While the EU used to be the biggest importer of soybeans, therefore allowing it some negotiating powers, China is now recognized as one of the biggest importers of soybeans (EU benefits from GM trade, EuropaBIo infographic).

Even in cases where a specific GM crop may not be envisaged as a product for a trading country, it is vital that the country's views and opinions should not negatively impact on other countries that stand to benefit from such technology. For example, Golden Rice, a product developed to provide an effective source of vitamin A to help address the serious health implications of vitamin A deficiency, including childhood blindness, has been approved in many countries for which a market is not foreseen. New Zealand and Australia approved this product for Food import in 2017, and Canada and the USA more recently in 2018 (ISAAA GM approval database). Here, the approval decision has been made to avoid potential lost trade should Golden Rice “contaminate” other rice imports, resulting in shipments being turned away. Other countries should follow suit if developing countries are to embrace the technology without fear of losing trade.

The ability of developing country farmers to produce higher yields (with reduced crop losses due to better weed and pest control) while lowering their input costs (labor and pesticide costs) is a significant factor in helping close the poverty gap (e.g., Ali & Abdulai, 2010; Kathage & Qaim, 2012). It is now estimated that biotech crops have been grown by 16–17 million small‐holder, resource poor farmers, and their families totaling >65 million people, in some of the poorest places in the world (Brookes & Barfoot, 2018).

There also remain a number of developing countries, especially in Africa, falling behind in establishing regulatory frameworks to enable their farmers to access the same technologies. It is estimated that these delays could, for example, have cost African agricultural economies US$2.5 billion from 2008 to 2013 due to lack of access to technology. The overall costs to the low‐ and lower middle‐income nations, in not having access to these technologies, are estimated to be up to US$1.5 trillion in foregone economic benefits through to 2050 (ISAAA, 2017).

## 5 GENOME EDITING: THE NEW REVOLUTION—HOW THE WORLD SEES IT VS HOW THE EU SEES IT

Genome editing (GE) is one of the so‐called “New Breeding Technologies” (NBTs) in the plant breeding tool box. It is of note to the reader that the abbreviation GE is used here for genome editing and should not be confused with the term genetic engineering which is used in some countries, outside of the EU, rather than the term genetic modification (GM). With regard to genome editing, there are many excellent reviews already available (e.g., (Songstad, Petolino, Voytas, & Reichert, 2017)) detailing the different types of GE techniques available, for example TALENS, ZFN, ODM, and CRISPR, with the latter increasingly leading the field. Clustered regularly interspaced short palindromic repeats (CRISPR) and CRISPR‐associated (Cas) nuclease 9 (CRISPR/Cas9) is allowing previously unimagined opportunities for precise genome editing. In this technology, a gene‐specific guide RNA (sgRNA) is designed and delivered along with the Cas9 nuclease to plant cells. This results in a double strand DNA break at a precise target location which is then repaired by the cell's own repair pathway (non‐homologous end joining, NHEJ) which sometimes results in an error leading to loss of function of the target gene. Variations on this technology allow single base changes to be made in target genes or for target gene expression to be up or down regulated. There are also categories of GE that allow for the insertion of new genetic sequence, and such crops would be regulated in line with the existing GMO regulations. However, it is the use of GE to create small indels or to create larger deletions within the existing genome that poses a new regulatory question. When GE is used to create a loss of function of a target gene, that is, contain no foreign DNA, should the end‐product be viewed any differently to products of conventional mutagenesis?

Many countries (e.g., Argentina, Brazil, Canada, Chile, Israel, the USA, and Japan) have already concluded that genome editing, in cases where new genetic sequences have not been directly inserted, that is, when the changes have been created by indels resulting from NHEJ repair or deletion of existing genetic sequence, should be no more regulated than a product of mutagenesis (Cluster, 2017; European Parliamentary Research Service, 2016; Kuzma, 2016; Smyth, 2017; Tetsuya & Motoko, 2017; USDA Press Release No. 0070.18). Chile and Brazil recently followed Argentina's lead, with Chile signing a normative resolution in 2017 and Brazil publishing a resolution in January 2018. Both countries will regulate gene‐edited products case‐by‐case and exempt them from regulation when there is no insertion of transgenes (Orozco, 2018). Meanwhile, in the EU some countries interpreted the 2001/18 EU directive on GMOs to suggest that in cases of genome editing, where the changes to the genome are similar to those resulting from conventional mutagenesis techniques, they should fall within the same exemption clause as laid out in Article 3 and Annex 1 of the directive. The first EU country to follow this path was Sweden with the Swedish Board of Agriculture considering a question from researchers in Umea and Uppsala about how to regulate five different lines of Arabidopsis, which had been created using several different techniques, including CRISPR‐Cas9, to produce plants (“mutants”) lacking a particular protein, PsbS, a so‐called safety valve in photosynthesis. While science treats the resulting plants as equivalent (they all lack the ability to produce PsbS), some techniques of producing the plants clearly fall within the scope of the GMO legislation while others fall outside, illustrating the problem with the current GMO definition. The Swedish Board of Agriculture deemed the mutations produced by CRISPR‐Cas9, to be exempt from the regulations once the introduced T‐DNA (containing the gene editing components) had been segregated away (“Green light in the tunnel”! Swedish Board of Agriculture, 2016). Indeed, this was the view the German Federal Office of Consumer Protection and Food Safety (BVL), when Canadian company Cibus approached them in 2014, to seek permission for field trialing their oligo‐directed mutagenesis (ODM) generated herbicide‐resistant canola. The BVL decided that this canola variety should not be considered a GMO. However, this decision was challenged in 2015 by several NGO groups and was left to the European Court of Justice to decide if certain classes of NBTs should be exempt from the EU GMO rules, and if so would this endanger the precautionary principle.

The European Court of justice (ECJ) delivered their final ruling on 25 July 2018. Before this date, in January 2018, the Advocate General provided the opinion that organisms obtained by mutagenesis are, in principle, exempt from the obligations in the Genetically Modified Organisms Directive. This opinion was suggested to apply to newer forms of mutagenesis as well as to older mutation breeding methods. Despite this legal advice, the final ruling from the ECJ (Court of Justice of the European Union, press release No.111/18) stated that “Organisms obtained by mutagenesis are GMOs and are, in principle, subject to the obligations laid down by the GMO Directive.” Only mutagenesis techniques conventionally used and with a long history of safe use (chemical and radiation mutation breeding) were exempt from these obligations. So, while new techniques of mutagenesis (such as genome engineering) can result in identical products, albeit obtained with more certainty and precision, these new technologies in the ECJ ruling were not considered to be exempt. As such they may be subject to the same regulatory burden and huge costs associated with GMO approvals. It will be interesting to see how Cibus will now view the EU market.

It could be argued that the question asked by the NGOs was more, should a herbicide tolerant (HT) crop be viewed differently depending on how it was developed? In this simpler scenario, the outcome is perhaps more understandable. Often the line between what the public is mistrusting of is blurred between that of GMOs and that of the use of herbicides; given most GM crops currently grown are HT. However, as technologies are evolving, the boundaries to what the EU is actually regulating are becoming blurred and questionable. If historically GM technology (i.e., the use of rDNA to introduce new genes into plants) has been shown to be no riskier per se than conventional breeding, should regulation move more toward consideration of the specific novel trait rather than the technology used to develop it? This is often referred to as a “product based” approval system, rather than a “process triggered” approval system (although this may be an over simplification. This argument is reviewed well in (Marchant & Stevens, 2015).

## 6 WILL HISTORY REPEAT ITSELF—COULD THE EU ULTIMATELY LOSE OUT BY DECIDING TO REGULATE CERTAIN CLASSES OF GENOME EDITED PLANTS AS GMOS?

While the recent ECJ ruling specifically sought to address the question on whether Cibus's herbicide tolerant canola should be exempt from the GMO regulations, the ECJ opinion will have far‐reaching consequences for scientific innovation in the EU, for the competitiveness of EU farmers, for industry and ultimately for consumers. The history of GMOs, the unfolding benefits to society, the environment, and its impeccable safety record have so far failed to persuade Member States to embrace the technology. This is likely to be an indication that the same fate may await GE products too.

A statement from the German Bioeconomy Council (September 2018) called upon policy‐makers to propose “a more differentiated assessment to modernise genetic engineering legislation.” Otherwise, fearing Germany “would remain out in the cold in this ‘biological revolution’ and would have no say in shaping the necessary international regulations” (Press Release from the German Bioeconomy Council). A similar response was given in an open letter to UK agriculture minister Michael Gove from 33 research institutions, universities, and plant breeders in the U.K asking for “a round‐table meeting involving all stakeholder, to agree a clear way forward on research and future use of new plant breeding technologies” (Call for clarity ‐ Open Letter to UK agricultural minister).

In choosing to follow a different regulatory path to the rest of the world, one can envision reduced optimism for these new technologies in the EU. This would result in investment again moving outside of Europe, due to the financial burden associated with regulatory approval (as seen with GMOs). Despite the 22‐year safe history of use, Europe has stubbornly adhered to its regulatory oversight. It has failed to make amendments and evolve the regulations as the evidence of safety of these crops grows. Indeed, there have been huge gains seen globally with the use of GM biotech crops to date. This together with a safe history of use, questions why the technology has faced so many barriers to adoption. Thus, we need to acknowledge that scientific safety and economic and environmental gains are not the only factors in play. Does the ruling by the ECJ potentially mean the benefits of GE could be lost before we have the chance to embrace them? Have the technologies that could have supported the organic movement (pesticide and fungicide free crops) been thwarted because being anti‐GM has been a great marketing tool. GE like GM has the potential to increase farmer profits and thus lower consumer prices, potentially widening the price difference between organic and non‐organic products and negatively impacting the organic market (Hamburger, 2018). Indeed, the organic sector has been quick to welcome the ECJ ruling (https://www.ifoam-eu.org/en/news/2018/07/25/press-release-new-genetic-engineering-techniques-be-regulated-gmos-ifoam-eu-welcomes).

At the same time, in response to the ECJ ruling, the European Seed Association (ESA) released a statement saying it considered the consequences of the ruling “to present unacceptable socio‐economic risks for European plant breeding, for the wider agri‐food chain, for consumers and for our European environment (European Seed Association ‐ statement). The prohibitive compliance requirements of the GMO 2001/18 Directive relative to the value of agricultural crops will effectively prevent European breeders taking advantage of these new technologies, putting breeders, farmers, processors, traders and consumers at a competitive disadvantage to regions with more enabling regulations.” The ESA highlight that plant varieties and seeds are already “subject to a respected and robust regulatory regime and those developed through the latest breeding methods should not be subject to different or additional regulatory oversight if they could be obtained through earlier breeding methods” (European Seed Association). An opinion echoed by the American Seed Trade Association (ASTA ‐ official statement on the ECJ ruling), who point out that the ECJ ruling is a legal interpretation of existing EU law, rather than a policy decision. “However, the court's interpretation contradicts the direction in which many other governments outside of Europe are moving, in respect to plant breeding innovation policy, and sets a dangerous precedent that could impede global trade and stifle innovation for the future… and the tremendous promise it holds for a more sustainable and secure global food production system.”

## 7 CONCLUDING REMARKS

It is clear the ECJ ruling has left many stakeholders baffled and seeking better clarity of the ruling and its implications. It will be of great interest to see how the decision affects Europe's momentum in research, and how an already failing regulatory system will keep up with the volume of products that will undoubtedly emerge from countries who choose to embrace new technologies. How crops developed using these technologies will be tracked through the international trade markets when there is no foreign DNA to detect is another major issue to resolve. Far from clarifying things, the ECJ decision leaves many questions still unanswered. With the urgent need to address both global food security and sustainability, genome editing offers the potential to make a significant contribution. Given the EU's policy commitment to assist developing countries in addressing food security challenges, especially in response to climate change (European Commission, 2010, EU policy framework to assist developing counties in addressing food security issues) it is time to acknowledge how the EU can better contribute to the long‐term goal of global food security, by implementing fit‐for‐purpose regulation of improved crops. Regulations need to be in harmony with the rest of the world to allow full exploitation of the potential of these new breeding technologies.

## CONFLICT OF INTEREST

None declared.
